# Dynamic Bayesian Networks for Context-Aware Fall Risk Assessment

**DOI:** 10.3390/s140509330

**Published:** 2014-05-23

**Authors:** Gregory Koshmak, Maria Linden, Amy Loutfi

**Affiliations:** 1 School of Innovation, Design and Engineering, Mälardalen University, Högskoleplan 1, Västerås 721 23, Sweden; E-Mail: maria.linden@mdh.se; 2 Center for Applied Autonomous Sensor Systems (AASS), Örebro University, Fakultetsgatan 1, Örebro 701 82, Sweden; E-Mail: amy.loutfi@oru.se

**Keywords:** ambient assisted living (AAL), fall detection, context recognition, multi-sensor fusion, dynamic Bayesian networks (DBN)

## Abstract

Fall incidents among the elderly often occur in the home and can cause serious injuries affecting their independent living. This paper presents an approach where data from wearable sensors integrated in a smart home environment is combined using a dynamic Bayesian network. The smart home environment provides contextual data, obtained from environmental sensors, and contributes to assessing a fall risk probability. The evaluation of the developed system is performed through simulation. Each time step is represented by a single user activity and interacts with a fall sensors located on a mobile device. A posterior probability is calculated for each recognized activity or contextual information. The output of the system provides a total risk assessment of falling given a response from the fall sensor.

## 1. Introduction

Aging populations have been one of the main concerns in most developed countries during the last decade [1]. Most elderly people suffer from a wider spectrum of various diseases, and more emergency situations, such as falls, are likely to occur [2]. As a result, they need to be transported to the hospital, observed and provided with medical help if their health condition is at risk. However, remote monitoring can help to prevent the described scenario, significantly reduce healthcare costs and, at the same time, maintain patient's independent lifestyle [3].

A growing trend in ICT is to combine monitoring components (e.g., sensors, actuators) into smart environments and carry out observations for people with multiple chronic conditions at home. Such systems are promoted in order to improve elderly patient's level of freedom and safety, which is one of the main issues in healthcare today. As fall injury is considered to be one of the most common and dangerous risks among the elderly population, it is reasonable that such fall detectors will also be integrated components in future smart homes. Today, the estimated fall incidence for both hospitalized and independently living people over 75 is at least 30% every year. Nearly half of nursing home residents fall each year, with 40% falling more than once [4]. These accidents can have both a physical [5] (often head injury) and psychological [6] (fear of falling) effect. Various methods have been proposed, including the smart home approach and wearable sensor monitoring. However, fall risk minimization is a complex problem and, therefore, requires a combination of measures to be applied.

In the current research, we deploy a wearable fall sensor in a smart home environment containing environmental sensors, merge the data into a single system and perform reliable fall detection. The approach adheres to information fusion, which is the process of dealing with a combination of information collected from disparate sources into one coherent structure, and can be deployed by a system to make better decisions than from single source [7]. In this paper, both components operate independently of each other, but are fused using a dynamic Bayesian network (discussed in Section 3.3). The premise of the proposed method is an isolated fall detection algorithm, which is based on an Android mobile phone and operates as a wearable sensor. It has additional functionality for capturing physiological measurements, such as pulse and the oxygen saturation of the patient. A smart home environment represents the second component of the system and covers various aspects of a patient's daily monitoring. In this case, we are particularly interested in contextual data collected from the RFID readers, pressure mats, *etc*. These sensor readings are further transformed into separate activities, performed by users, fused with wearable sensor data and processed, resulting in a context-aware fall detection system. Work on the detection of the activities is presented in [8]. This paper's contribution is in the sensor fusion method implemented through a dynamic Bayesian network, which is commonly used for time series and statistical data.

This work is part of the collaborative project, GiraffPlus (the study was partly financed by the EU-FP-7 project, GiraffPlus, and the Knowledge Foundation's research profile, Embedded Sensor Systems for Health (ESS-H)), which is focused on independent living for elderly people. As an example of an ambient assisted living (AAL) system, GiraffPlus implies various types of functionalities, including continuous monitoring, alarm generation or direct communication with remote caregivers [9]. It contains a combination of environmental and physiological sensors intended to capture most of the patients' activities and changes in lifestyle or health conditions. All the data are stored in the global database through a special middleware, connecting different components of the system. GiraffPlus system is a multi-functional model that can cover different types of monitoring scenarios and also includes a focus on fall detection.

The rest of this paper is organized as following. We start with related work, describing the latest developments in wearable sensors, smart home systems and their role in the fall detection area. We proceed with the main framework and system overview, where we present a sensor fusion algorithm based on a dynamic Bayesian network. This part is followed by evaluation work and consequent results. Finally, we conclude with the system functionality and future perspectives in Section 6.

## 2. Related Work

### 2.1. Context-Aware Fall Detection

Smart homes are part of the ambient assisted living area, responsible for the continuous monitoring of elderly people in a comfortable home environment. In recent years, an increasing number of projects have been based on this approach utilizing various components and application purposes. For example, a special nutrition advisor was proposed as an attempt to improve the physical condition of elderly people with diabetes [10]. It demonstrates the possibility to improve the efficiency of nutritional management through deploying ambient intelligence systems. Moreover, Juan A. Botia, Ana Villa and Jose Palma presented their Necesity system, with adaptive monitoring capabilities and an exhaustive evaluation methodology that were integrated in the development process [11]. Researches in the eCAALYX project (Enhanced Complete Ambient Assisted Living Experiment) take 24/7 monitoring of healthy older people one step further by refining and making it available to older people with multiple chronic disease. A particular effort is made in communication with the user, deploying various sorts of interactive devices: a TV-based set top box system, customer-premises equipment and interactive TV [12]. It becomes clear that smart environments equipped with various sensors can potentially cover a significant number of health issues, providing essential information about patients' life status.

In the current research, however, we are particularly interested in deploying contextual data for accurate fall detection. A similar approach was described in the article by Brulin *et al.* [13], where the main idea is to fuse different types of data source channels into a special architecture, which allows us to ensure a constant acquaintance of different information from Passive Infrared (PIR) detectors, thermopile or cameras. This developed system also performs on-line or posterior processing to derive the posture or orientation of the user and triggers an alarm in case of fall risk. Various attempts were made to improve this process by introducing additional sources of information, like surrounding audio captured by microphone arrays [14,15] or the current location of the user [16]. Alternatively, an RGBD-camera was deployed in the study by BinBing Ni *et al.* [17] for hospital fall prevention. To prevent potential falls, once the event of ‘patient gets up from the bed’ is automatically detected via a Microsoft Kinect sensor, nursing personnel is alarmed immediately for assistance. However, the contextual data approach lacks accelerometer measurements captured directly from the patients body, which makes the system incomplete. In a recent effort by Ferreira *et al.* [18], a fall prevention system makes use of collected data from sensors in order to control and advise the patient or even to give instructions to treat an abnormal condition to reduce the fall risk. Monitoring and processing data from sensors is performed by a smartphone that will issue warnings to the user and, in grave situations, send them to a caretaker. Moreover, the relationship between the acceleration of the body's center of gravity during sit-to-walk motion and the process of falling is investigated by Shiozawa *et al.* [19]. The result of discriminant analysis by using indexes with a significant difference revealed a 90.3% correct prediction rate for falling.

Wearable sensors with an inbuilt accelerometer can play an essential role as a complementary component for smart home environments and have recently been deployed for accurate fall detection [20]. In our case, a wearable device is replaced with a smartphone, which can serve as an accelerometer sensor and the gateway mode at the same time.

### 2.2. Mobile Healthcare Integration

With the recent development of the mobile market, smartphones are beginning to play an important role in modern healthcare systems [3]. New features create new opportunities to use smartphones or tablets for managing and presenting medical data. The list of possible applications has been growing along with market development: early detection of Alzheimer's disease [21], face-to-face communication between doctor and patient [20], complex activity recognition [22] and medicine intake assistance [23]. Modern smartphones equipped with an accelerometer sensor are also commonly used as fall detection tools [24–27]. They replace both the processing mode and a communication gateway, while maintaining a relatively small size. The choice of processing algorithm depends on the final application of the system and varies in different studies. Some of the recent implementation methods apply Gaussian distribution of clustered knowledge [28], neural network [29] and machine learning techniques [30]. However, most are initially based on three essential parameters associated with falls: impact, velocity and posture. According to a recent article, combining impact and posture, while analyzing the fall incident, is enough to create a reliable algorithm [31].

In our research, we deploy an isolated fall detection system, implemented on the mobile device as an element of the integrated smart home environment. A similar approach was adopted in several studies with the intention of combining contextual data with essential accelerometer measurements exploiting inertia and location sensors [32]. Qiang Li *et al.* [33] investigated a novel fall detection method that utilizes acceleration, posture and context information, where context can be presented by environmental sensors (room location or furniture positions) and personal profiles (e.g., health status and age). A wireless accelerometer, 3-D camera and microphone are simultaneously processed by Leone *et al.* to reach a better result in fall risk assessment [34]. All the presented studies, however, are lacking a reliable fusing technique, combining processing results from independent components. In work by Zhang et al. [35], an off-the-shelf programmable sensing platform, called Sun SPOT, is used for data recording. Context information is presented in several categories, covering the main aspects of elderly living: (1) physical activity; (2) physiological condition; (3) personal health record; and (4) location. Each category represents a separate variable or processing result (including a fall alarm from the isolated algorithm) and merged into a Bayesian network for further statistical analyses. The system's output represents a probability of the fall to occur, given the contextual information. The formulated approach provides a convenient tool for managing different data channels, but fails to consider time reference in the process. In many cases, most of the system elements directly depend on the their previous states, which should be taken into account. This requirement can be covered by introducing an extension to regular probabilistic networks commonly addressed as dynamic Bayesian network, a statistical model, which has been previously deployed in a number of studies, including sensor fusion vehicle localization and road matching [36,37]. In Section 3, we show the method of managing multi-variant healthcare data with a dynamic Bayesian network and describe the framework of the developed system.

## 3. Framework

### 3.1. Mobile-Based Fall Detection System

**Figure 1.**
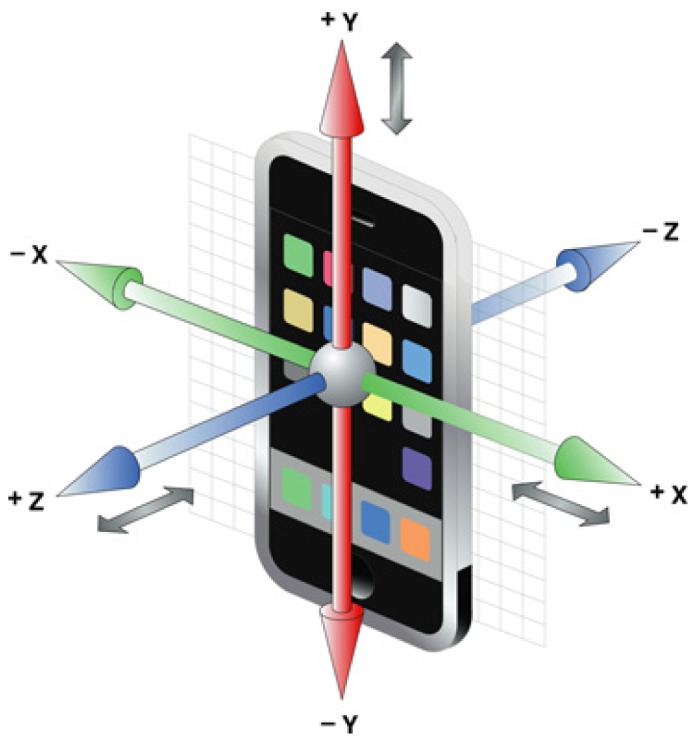
Smartphone coordinate axis.

The first component of the integrated platform is the isolated fall detection system, which is implemented on the Android-based mobile device and operates as a wearable sensor. In our previous study [38], we deployed embedded a three-axis accelerometer, depicted in Figure 1, and designed a multi-functional application based on the Android operating system device. This application is able to carry out both activity and physiological data monitoring, with more than one process running simultaneously on the phone. Therefore, a simple, yet sufficient, algorithm with a low power consumption rate was created to satisfy these constraints.

The mobile device is located on the central part of the waist, corresponding to the body's center of gravity. For maintaining the acceleration axis, the device is fixed in a special phone case attached to the user's belt. The process is split into three main stages. The system starts to receive data from the accelerometer and calculates the overall acceleration value Act:

$$\text{Act}=E[\left|\nu_{a}^{2}-E[\nu_{a}^{2}]\right|];\nu_{a}=\sqrt{a_{x}^{2}+a_{y}^{2}+a_{z}^{2}}$$

where *a_x_, a_y_, a_z_* correspond to acceleration along three axes of the smartphone coordinate system. If an impact is registered (*Act* ≥ threshold) during the activities of daily living, we consider it a potential fall risk, pause the monitoring process and check the orientation of the phone. Using the same acceleration values, *a_x_, a_y_, a_z_*, we determine the device's Euler angles with respect to the Earth's gravitational attraction:

$$\begin{array}{c} \text{roll}=\text{asin}(\frac{a_{x}}{\text{gravity}})\\ \text{pitch}=\text{asin}(\frac{a_{z}}{\text{gravity}}) \end{array}$$

A similar approach is used in games or bubble-level applications for smartphones [39]. Unlike methods utilizing only registered impact in acceleration, the developed algorithm deploys the current orientation of the body, which allows one to avoid alarms during high-impact activities (e.g., sitting in a chair, stumbling, rising out of bed, *etc.*). A combination of thresholds for roll and pitch angles corresponding to the horizontal position of the body triggers an alarm signal informing the user about the fall. Before forwarding this message to a smart home database, the algorithm complements an alarm with additional data. First, we deploy accelerometer values occurring during the impact in order to determine the fall's direction (forward, backward, left side, right side). Each direction corresponds to a particular combination of acceleration numbers along the axis (see Figure 1). Furthermore, physiological measurements are collected during the monitoring process as a complementary unit. Every time the system detects a fall, the last pulse and oxygen saturation value is attached and sent along with the person's location and fall direction number. The combination of the additional data can vary depending on application purposes and current set-up. The evaluation process in the fall detection area is based on a common approach, where young and healthy volunteers are asked to simulate falls multiple times in different directions [40]. We found a fair solution, which evaluates the system in a real environment and contains both regular movements and fall incidents. After personal consent, we asked seven volunteers with minor winter sports skills to wear the mobile fall detection system while performing ice-skating activity. Due to the lack of experience in this sport, falls occurred consistently, together with frequent and random motions of the torso. The proposed approach helps to test the system in situations close to real life when falls appear unintentionally. Besides, a fair amount of physical activity is involved in ice-skating, which can be considered as a stress test to avoid false positive results. All the volunteers performed from 15 up to 30 min of ice-skating during each session, which resulted in 50 falls overall. No participant was injured, and the experimental part finished successfully. The evaluation process resulted in 90% sensitivity, 100% specificity and 94% accuracy of the developed algorithm.

However, these results cannot be entirely objective assuming that the final target users of the current project are elderly people. In this case, we have to deal with a different level of body motions, a higher risk of false positive alarms and data misinterpretation. Therefore, in order to guarantee the reliability of the final system and to account for the mentioned constraints, we integrated contextual data, collected in the smart home environment, in the common framework.

### 3.2. Context Recognition

In our case, contextual data are represented by a set of sensors located throughout an apartment, including PIRmotion, door contact, pressure mats and power usage detectors. A special context recognition system, designed and described by Ullberg *et al.* [41], infers human activities from raw sensor data. The context recognition model consists of several independent modules, including preprocessing, inference and extraction modules.

The preprocessing module fetches samples from the sensors located in the environment and builds a higher lever representation of the events that take place at home. For instance, a timestamped series of temperature readings can be “down-sampled” to a single or a set of temporal intervals, indicating that the temperature was “high” during the corresponding periods of time. The preprocessing is the only module that fetches samples from the remote database [42].

The inference module infers activities from the intervals, previously generated by the preprocessing module. For instance, it can deploy the data coming from the temperature sensor mounted to a warm-water pipe to deduce that the inhabitant is showering. In this hypothetical case, a rule could define showering as an activity that occurred during “In_Bathroom”and contains “Temperature_High_Shower”.

**Figure 2.**
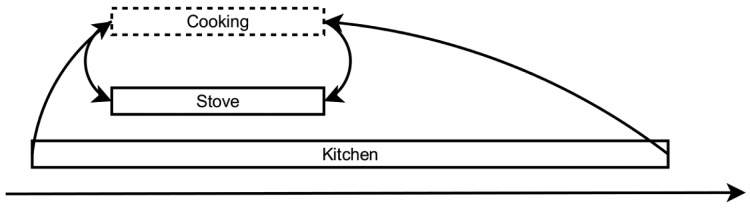
Inferring “cooking” activity.

Finally, the extraction module is responsible for generating time-lines that can be visualized for the user. Currently, this module supports only one type of extraction method, which simply extracts the maximum duration interval for an activity. We can illustrate a context recognition process on a simple example of how the activity of “cooking” can be inferred from intervals representing sensed data (see Figure 2). In order to infer the context, the generated set of intervals is combined with a model consisting of rules of the form:

Cooking=Stove∧CookingDuringKitchen

These types of rules define abstract patterns of constraints in Allen's interval algebra [43]. The final output of the context recognition block is a set of activities of daily living (ADL), which are subsequently merged together with the isolated fall detection algorithm results (described in Section 3.1). We deploy a dynamic Bayesian network and design a specific system, configured according to our case, to perform multi-sensor fusion.

### 3.3. Dynamic Bayesian Network

**Figure 3.**
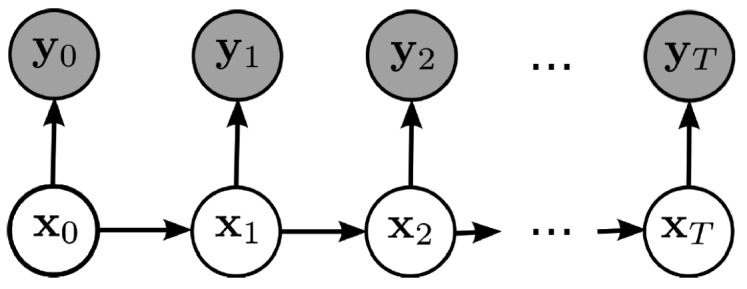
Hidden Markov model (HMM).

As was previously mentioned in Section 1, information fusion implies the combination, association and correlation of multiple data sources for better decision-making. Generally, this process can be described with the following formula:

$$\theta =F(S_{1},S_{2},\ldots ,S_{n})$$

where *S_i_* denotes disparate sensor readings, *F* is a fusion function responsible for merging data and *θ* represents the final output utilized for decision-making [7]. In our particular case, a dynamic Bayesian network (DBN) is deployed as a fusion method with probabilistic inference as a fusion function. The output, *θ*, is the posterior probability of the fall risk, which we try to infer. Unlike standard probabilistic networks, DBNs are suitable for modeling dynamic events and are represented by directed graphical models of stochastic processes, which are defined by means of a directed acyclic graph (DAG) as follows:

$$P(X_{t}|Y_{t-1})=\prod_{i=1}^{T}P(X_{t}^{i}|\pi (Y_{t}^{i}))$$

where *X*(*i, t*), *Y* (*i, t*) represent hidden and observed nodes (variables) accordingly. A DBN model is made up of two interconnected time slices of a static BN, with parent nodes *Pa*(*Y*_(_*_i_*_,_*_t_*_)_), either at time slice *t* or *t* − 1 and the transition of BN, which satisfies the Markov process. The simplest kind of DBN is a hidden Markov model (HMM), which has one discrete hidden node and one discrete or continuous observed node per slice. It is depicted in Figure 3, where clear circles denote hidden layers and shading represents observed nodes.

The model is unrolled for three initial time slices, but the structure is assumed to repeat as the system is unrolled further. Normally, to specify a DBN, we need to define the intra-slice topology (connections between variables within one slice) and inter-slice topology (between variables from different slices), as well as parameters for the first two slices. The most common task within the Bayesian networks is to perform probabilistic inference using Bayes theorem; $P(A|\text{B})=P(A)\frac{P(\text{B}|A)}{P(\text{B})}$. At the same time, the graphical model mentioned above also specifies a complete joint probability distribution (JPD) over all of the variables. Given the JPD, we can answer all possible inference queries by marginalization (summing out over irrelevant variables). In the case of using DBN, the general inference problem is to compute *P*(*X*(*i, t*0)|*y*(:, *t*1 : *t*2)), where *X*(*i, t*) represents the *i*–th hidden variable at time *t* and *Y* (:, *t*1 : *t*2) represents all the observed variables ( evidence) between times *t*1 and *t*2. In other words, we try to calculate the current state of hidden variable, given the evidence. Several special cases of interest can be used for this purpose, including filtering, Viterbi, prediction, fixed-lag smoothing and fixed interval smoothing. A final effort is made to determine the posterior probability representing a fall risk for subsequent decision-making. In the following section, we describe the representation and inference in the DBN in our particular case of multi-sensor fusion for home monitoring.

## 4. System Integration

**Figure 4.**
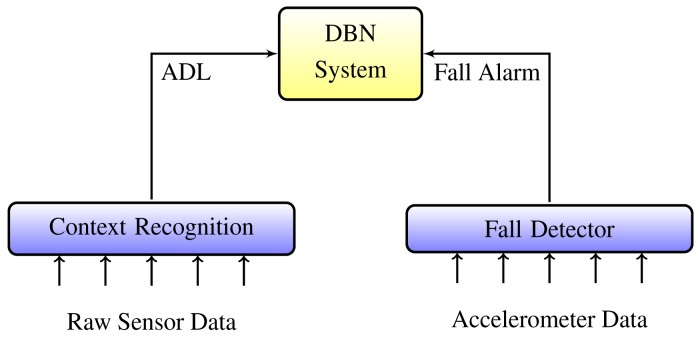
The integrated system. DBN, dynamic Bayesian network; ADL, activities of daily living.

Two independent components of the system described in Section 3 represent different source channels and provide incompatible outputs (see Figure 4). The wearable sensor operates on accelerometer data and generates alarms based on fall impact and orientation angles. Context recognition retrieves data from environmental sensors and infers regular ADLs.

We deploy a statistical method described in Section 3.3 and combine both components into a single platform, based on a dynamic Bayesian network. In this case, each activity derived from the context or fall detection algorithm is represented by specific variables and comprises the evidence of the network. A preliminary list of ADLs that can be performed by the elderly and be detected by context recognition is introduced in Table 1 and contains: (1) SL, sleeping; (2) Sh, taking a shower; (3) C, cooking; (4) TV, watching a TV; (5) Tr, training, physical exercise; and (6) FA, fall alarm.

One extra variable is derived from the isolated fall detection algorithm and included in the system as FE (fall event). In this way, we transform an alarm, based on accelerometer data, into a statistical unit and merge it with the rest of the system. In a similar way, we are able to introduce another variable based on physiological data collected from the medical sensor. Nevertheless, the research work in the GiraffPlus project is still in progress and a final list of activities is yet to be confirmed. The flexibility of the system, however, allows us to adjust parameters and include new variables as they are confirmed. Based on the outlook of the integrated platform, it is possible to establish the essential goal of the studies, which implies estimating fall risk probability or, in other words, calculating FA.

**Figure 5.**
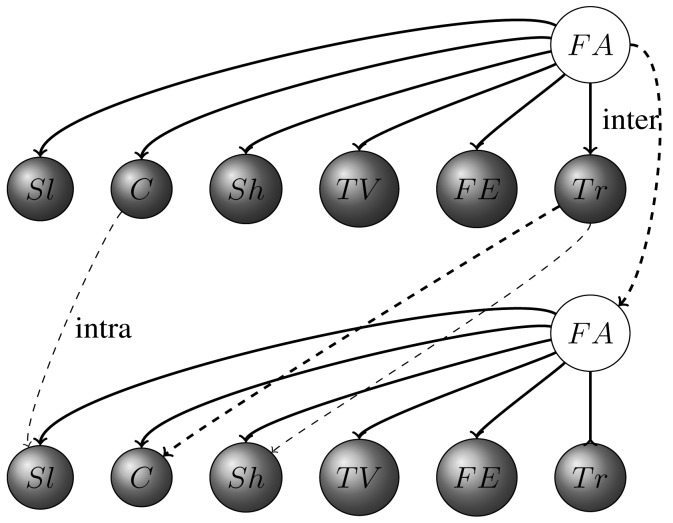
The system's directed acyclic graph (DAG) unrolled for two time slices.

As the initial step, we deploy the MATLAB DBN toolbox [44] provided by Kevin Murphy for building a statistical model. Commonly, DBN consists of the graph structure (visualized in Figure 5) and parameters (see Table 2). Following the standard approach specified in the hidden Markov model DAG, shaded circles represent observed variables, whereas clear ones are hidden nodes. As an initial step, we define all the intra- and inter-connections between each element within the network. Once the connectivities are set, we adjust parameters for the listed variables before executing the inference process.

The standard procedure implies forming simple conditional probability tables (CPT), which define the probability distribution of a node (e.g., random variable) given its parents. In MATLAB CPTs are stored as multidimensional arrays, where dimensions are arranged in the same order as the nodes. In this way, conditional tables for Sl (see Table 2), for instance, are first indexed by FA and then Sl itself. Hence, the child is always the last dimension. If a node does not have a parent (as node FA in our case), the CPT is represented by the prior vector containing the initial probability distribution. We also make a convention that false = 1 and true = 2, which makes a simple example of the conditional dependency for Sl activity look as follows: *Pr*(*Sl* = *true*|*FA* = *true*) = 0.1. This equation corresponds to common life experience, indicating an extremely low chance of falling while the patient is asleep.

Unlike simple Bayesian networks, DBN can contain variables that are inter-connected with their parents. In this case, we introduce additional CPT (see Table 3) for the nodes from the second time slice, which are modified based on the conditional dependencies between variables. In the following example, Sh activity is indexed by Tr, FA and then by Sh itself. The main challenge while forming CPTs is to assign the correct probability values for each variable. There are no precise statistical data covering the probability of falling, given that the person is performing any of the corresponding ADLs. We can consider a simple approach and assume that any frequent movement implies a higher chance of falls to occur. In other words, if the person is training, there is a higher risk for falling than if the same person is watching a TV. Therefore, we can utilize the accelerometer data and calculate the probabilities for each activity in relation to an initially set value. This approach, however, is not taking into account patients' personal information, and the parameters may vary from one individual to another. Alternatively, it is possible to deploy DBN learning features and to perform system learning for parameter adjustment. In this way, we will be able to assign CPT values based on previous experience, which is considered as the future work of studies.

Once the graphical structure and parameters are set, we can proceed with inferencing and use a previously developed network to find an updated state of the hidden variable subset, when other variables are observed.

In DBN, this also means computing the probability *P*(*X*(*i, t*0)|*y*(:, *t*1 : *t*2)), where *X*(*i, t*) represents the *i*-th hidden variable at time *t* and *Y* (:, *t*1 : *t*2) represents the evidence between times *t*1 and *t*2. If all the hidden nodes are discrete, as in our case, it is reasonable to apply the junction tree algorithm to perform inference. In order to avoid numerical underflow and decreased processing time, jtree is applied to pairs of neighboring slices at a time; this is implemented in *jtree_dbn_inf_engine* from the same DBN toolbox [39].

### 5.1. MATLAB Simulated Model

**Figure 6.**
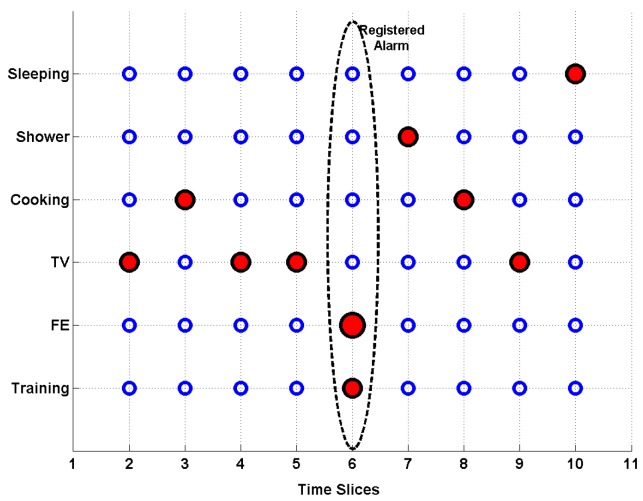
Simulation process.

After creating a statistical model, we can perform the evaluation step, which is split into the simulation and demonstration part. In the first case, we deploy MATLAB functionality and unroll a previously created model for 100 time slices (the first 11 slices are shown on Figure 6) to simulate the monitoring process.

This approach allows us to test the system in terms of a continuous monitoring process and check its efficiency in avoiding the actual risk of falling. We are not interested in fall mechanics or the health conditions of the patient, but the fall frequency and system reactions instead. Our goal is to show the flexibility of the algorithm regardless of the monitoring object or test environment. Therefore, all the elements and parameters of the model can be easily removed or adjusted depending on system configurations and the current monitoring state.

The choice of time slice unit is entirely subjective and can represent one minute, one hour or several hours, depending on current purposes and monitoring circumstances. Every slice contains a number of activities that could happen simultaneously during this period of time, including a fall event or every possible ADL. Our simulation scenario generates one single activity from context recognition per time unit with the fall event interfering every 10th time slice. In other words, every time step, we obtain only one observed variable and seven hidden nodes, which correspond to a system with a known structure, but partial observability. In this case, we can use the expectation maximization (EM) algorithm to find a (locally) optimal maximum likelihood estimate (MLE) of the parameters. Therefore, each step, we compute the expected values of all the nodes using an inference algorithm and, then, treat these anticipated values as though they were observed (distributions). As previously mentioned, the simulation method can help us to recreate continuous monitoring and avoid health risks during the experimental part of the studies. Nevertheless, it is technically randomized and, therefore, cannot completely correspond to the real-life scenario.

We propose an alternative approach representing the monitoring procedure to overcome the random aspect of the MATLAB simulation process. It implies a short system demonstration, where raw acceleration data are collected, while a healthy volunteer performs regular ADLs.

**Figure 7.**
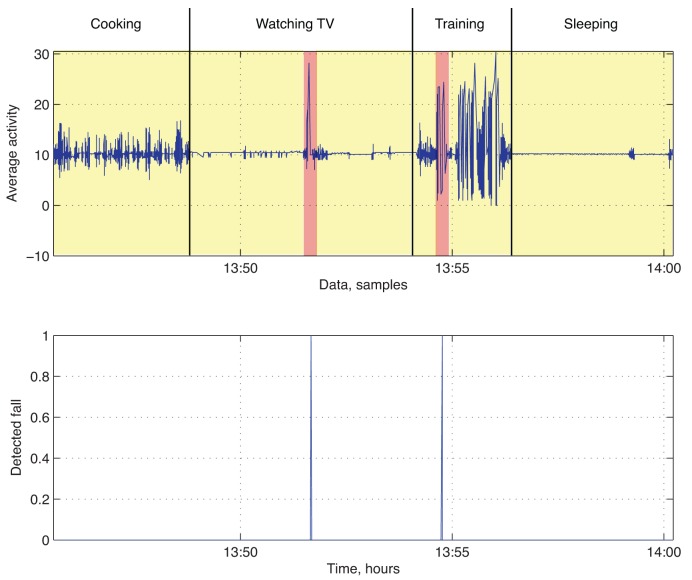
System demonstration.

These data are subsequently deployed as the ground material for the evaluation process. Unlike the simulation method, all the activities are performed according to natural behavior and, therefore, allow us to avoid unrealistic sequences. In total, four different ADLs were executed during a short-term demonstration with two fall alarms triggered by the mobile phone-based algorithm, which is depicted in Figure 7. The first fall was registered during the “watching TV” activity (see Table 1) and was supposed to represent a false positive alarm. A similar scenario is likely to occur if the mobile device is exploited inappropriately by the patient or a technical error is affecting the algorithm. Therefore, it is essential to take into account context information and check the current activity of the user. The second (actual) fall was registered during the “training” activity and was performed by a volunteer without any health issues. After short- term data collection and minor preprocessing, we deploy MATLAB functionality and proceed with sensor fusion.

As a result, from both evaluation scenarios, we obtain the full observability of the system, indicating the probability of every event registered in the network for a particular time slice. However, our main interest is fall risk assessment, and therefore, it is essential to calculate $P(FA|\text{ADL})=P(FA)\frac{P(\text{ADL}|FA)}{P(\text{ADL})}$, the probability of FA, given any ADL, using the Bayes rule from Section 3.3; or, in the case of activity overlap, when more than one node becomes observed, we compute *Pr*(*FA*|*C, TV, Tr, Sh, Sl, FE*), which is also known as “explaining away” or Berkson's paradox in statistics [45]. Moreover, computing the fall risk associated with context information and the isolated fall detector is not the only option provided by the system. We are able to estimate the probabilities for each event to occur, based on previously observed data, which gives an opportunity to perform parameter learning.

### 5.3. Fall Risk Probability Estimation

**Figure 8.**
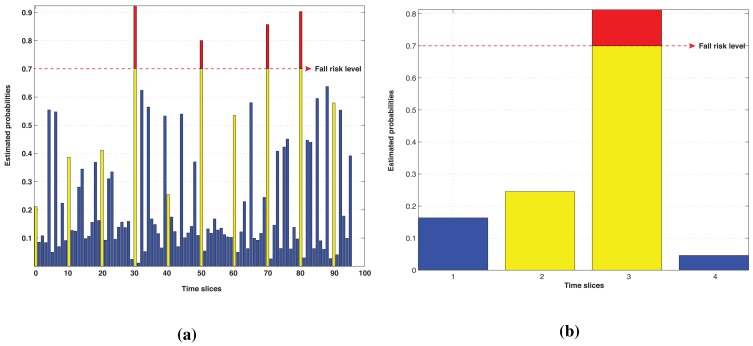
Evaluation results. (**a**) Simulation results; (**b**) Demonstration results.

The final step of the algorithm evaluation process implies calculating the FA probability value for each time step after performing the simulation and demonstration scenario. The estimated result after the MATLAB simulation process is summarized in Table 4. The outcome from both evaluation processes is depicted as a bar graph in Figure 8, where blue components represent the regular time slices and every 10th element (in yellow) demonstrates how the probability is affected by the FE activity.

We also set a fall risk level to indicate potential emergency situations. This level is a flexible parameter, which can be adjusted depending on the current user or monitoring scenario. According to the developed configuration, the integrated system manages only activity data, obtained from the context recognition module. At this stage, the inference algorithm provides probability estimation for the set of activities, based on their previous values and current configuration.

Certain activities depend on their previous state or the previous state of their interconnected parent, which is reflected in corresponding conditional dependencies and specified in conditional probability tables (see Tables 2 and 3 in Section 4). For instance, in our case, “sleeping” is likely to occur right after “shower” (*Sh* → *Sl* in Figure 5) and “shower” and “cooking” after “training” (*Tr* → *Sh, Tr* → *C*). Besides, it is also highly unrealistic that two falls happen consequentially after each other, which is covered by the *FA* → *FA* connection.

Whenever FE activity is received, it implies that the physiological sensor registered an alarm. Subsequently, the DBN performs the multi-sensor fusion process, takes into account previously collected contextual data and calculates the final output based on both components of the monitoring system.

In the case of the simulation process, 10 alarms were received from the fall detector located on the phone, and only four of them were confirmed by the system. The list of confirmed emergency cases is laid out in Table 5 together with the probability outcome and neighboring activities. In this way, we are able to eliminate all the false positive alarms generated by the wearable sensor (including alarms triggered during “showering” and “sleeping” activities) and improve the level of reliability of the fall detection algorithm.

After processing the data collected during the demonstration, we performed a similar procedure for calculating the posterior probability of the fall risk. In this case, two alarms were subsequently triggered by the independent fall detector, and one of them was canceled after applying the DBN-based multi-sensor fusion. The first fall occurred during the “watching TV” activity and was faked by the user. Therefore, it received a low probability value and was classified as a false positive. The second alarm was registered during the training activity and represented an actual fall. It was confirmed by the system and can be subsequently processed and used to notify a caretaker. Apart from that, various sources of data combined into a single system provide different types of measurements and, therefore, processing opportunities for future research, which is discussed in Section 6.

## 6. Conclusions and Future Work

The initial goal of the studies implied the combination of various sensors based on a single platform to provide reliable and stable fall detection. We were able to deploy a phone-based wearable sensor together with contextual data and fuse the results from both sources based on a dynamic Bayesian network. This paper is an attempt to explore the benefits of applying a statistical method for the continuous monitoring of the physiological and contextual information for elderly home care. Therefore, the structure of the network corresponds to the predefined monitoring scenario, and the parameters are set according to subjective assumptions.

The evaluation part of the study demonstrates that the suggested multi-sensor fusion approach is applicable for on-line monitoring and fall detection, disregarding the sensor features involved in the process. Assuming the flexibility of the algorithm, it can be equally applied to different sources of data after the simple adjustment of the appropriate parameters or introducing supplementary variables. With the current set-up, we were able to detect false positive alarms received from the isolated fall detection algorithm and provide thorough statistical information regarding the fall risk probability for the user.

We also consider a number of improvements, which can be addressed as the future work of these studies. First, the possibility implies investigating the learning capabilities of dynamic Bayesian networks. Using its broad functionality, we will be able to assign CPT values automatically, detect similar patterns during monitoring and predict different types of alarms based on these data. Another opportunity to enhance the system can be found in deploying physiological data received from the medical sensors. In this case, professional doctors will be able to see correlations between the daily activity of the person, the amount of movement and essential health parameters. We believe this approach leads to a better understanding of the cause, nature and the consequences of falling among the elderly.

## Figures and Tables

**Table 1.** Preliminary list of activities.

**Activity**	**Abbreviation**	**Details**
Sleeping	Sl	pressure bed sensor + presence in the bedroom
Showering	Sh	tap sensor + presence in the bathroom
Cooking	C	stove sensor + presence in the kitchen
TV watching	TV	TV sensor + presence in the living room
Fall Alarm	FA	final indication of the fall
Training	Tr	exercise machine sensor + accelerometer sensor
Fall Event	FE	fall indication received form the phone-based detector

**Table 2.** Conditional probabilities.

	
**Sl**	**FA**	**Probability**	**TV**	**FA**	**Probability**
	
1	1	0.4	1	1	0.5
2	1	0.6	2	1	0.5
1	2	0.9	1	2	0.87
2	2	0.1	2	2	0.13
	

**Table 3.** Conditional probabilities, including interconnections.

	
**Tr**	**FA**	**Sh**	**Probability**	**Tr**	**FA**	**C**	**Probability**
	
1	1	1	0.9	1	1	1	0.6
2	1	1	0.2	2	1	1	0.2
1	2	1	0.9	1	2	1	0.6
2	2	1	0.5	2	2	1	0.6
1	1	2	0.5	1	1	2	0.4
2	1	2	0.8	2	1	2	0.8
1	2	2	0.1	1	2	2	0.4
2	2	2	0.1	2	2	2	0.4
	

**Table 4.** Simulation process summary.

**Activity**	**Amount**	**min. FA**	**max. FA**	**Average**	**Alarms**
**Sh**	28	0.01	0.53	0.14	0.2, 025, 0.53
**Sl**	24	0.05	0.2	0.14	0.38, 0.4, 0.6
**C**	17	0.09	0.8	0.29	0.8
**TV**	12	0.03	0.23	0.13	-
**Tr**	18	0.24	0.92	0.58	0.85, 0.9, 0.92

**Table 5.** List of confirmed alarms.

**Time Slice #**	**Formula**	**Probability**	**Previous event**	**Following event**
30	*P*(*FA*|*Tr*)	0.92	**Sh**	**Sh**
50	*P*(*FA*|*C*)	0.79	**C**	**Sh**
70	*P*(*FA* |*Tr*)	0.85	**Tr**	**Sh**
80	*P*(*FA*|*Tr*)	0.89	**C**	**TV**
